# Progranulin derivative Atsttrin protects against early osteoarthritis in mouse and rat models

**DOI:** 10.1186/s13075-017-1485-8

**Published:** 2017-12-19

**Authors:** Jian-lu Wei, Wenyu Fu, Yuan-jing Ding, Aubryanna Hettinghouse, Matin Lendhey, Ran Schwarzkopf, Oran D. Kennedy, Chuan-ju Liu

**Affiliations:** 1 Department of Orthopaedic Surgery, New York University Medical Center, New York, NY 10003 USA; 2 Department of Orthopaedic Surgery, Qilu Hospital, Jinan,, Shandong 250012 China; 3 Department of Cell Biology, New York University School of Medicine, New York, NY 10016 USA; 4 Rm 1608, HJD, Department of Orthopaedic Surgery, New York University Medical Center, 301 East 17th Street, New York, NY 10003 USA

**Keywords:** Atsttrin, Progranulin, TNFα, TNFR2, TNFR1, Osteoarthritis

## Abstract

**Background:**

Atsttrin, an engineered protein composed of three tumor necrosis factor receptor (TNFR)-binding fragments of progranulin (PGRN), shows therapeutic effect in multiple murine models of inflammatory arthritis . Additionally, intra-articular delivery of PGRN protects against osteoarthritis (OA) progression. The purpose of this study is to determine whether Atsttrin also has therapeutic effects in OA and the molecular mechanisms involved.

**Methods:**

Surgically induced and noninvasive rupture OA models were established in mouse and rat, respectively. Cartilage degradation and OA were evaluated using Safranin O staining, immunohistochemistry, and ELISA. Additionally, expressions of pain-related markers, degenerative factors, and anabolic and catabolic markers known to be involved in OA were analyzed. Furthermore, the anabolic and anti-catabolic effects and underlying mechanisms of Atsttrin were determined using in-vitro assays with primary chondrocytes.

**Results:**

Herein, we found Atsttrin effectively prevented the accelerated OA phenotype associated with PGRN deficiency. Additionally, Atsttrin exhibited a preventative effect in OA by protecting articular cartilage and reducing OA-associated pain in both nonsurgically induced rat and surgically induced murine OA models. Mechanistic studies revealed that Atsttrin stimulated TNFR2-Akt-Erk1/2-dependent chondrocyte anabolism, while inhibiting TNFα/TNFR1-mediated inflammatory catabolism.

**Conclusions:**

These findings not only provide new insights into the role of PGRN and its derived engineered protein Atsttrin in cartilage homeostasis as well as OA in vivo, but may also lead to new therapeutic alternatives for OA as well as other relative degenerative joint diseases.

**Electronic supplementary material:**

The online version of this article (doi:10.1186/s13075-017-1485-8) contains supplementary material, which is available to authorized users.

## Background

Osteoarthritis (OA) is a degenerative joint disease characterized by cartilage destruction, synovitis, subchondral bone sclerosis, and osteophyte formation; OA affects almost 15% of the world’s population [1]. Unfortunately, the mechanisms of OA development remain unclear and there are no available therapeutic agents that effectively prevent or arrest progression of the disease [2, 3]. Although the etiology of OA is still unclear, it is believed that tumor necrosis factor alpha (TNFα) exhibits an important effect in the pathological processes of OA [4]. The level of TNFα is increased in OA patients’ articular cartilage compared with that of healthy controls and TNFα is thought to cause inflammatory destruction [5]. Additionally, anti-TNFα drugs demonstrate preventative and therapeutic effect in various OA models as well as in clinical trials [6].

Progranulin (PGRN) is a growth factor with a unique “beads-on-a-string” structure [7, 8]. PGRN participates in many pathophysiological processes, including anti-inflammation, tissue repair, and wound healing [9–12]. Importantly, PGRN is also a growth factor involved in regulating cartilage development and degradation, and the PGRN level is significantly increased in OA patients’ cartilage relative to that of healthy controls [13, 14]. Previously, we found that PGRN binds to tumor necrosis factor receptors (TNFRs) and is therapeutic in multiple mouse models of inflammatory arthritis [6, 15]; our recent studies, implementing surgically induced OA models, reveal that PGRN also protects against OA through TNFR signaling [16].

Through screening a series of PGRN deletion mutants retaining TNFR binding ability, we have generated an engineered protein which appears to be the minimal molecule of PGRN that still has TNFR binding affinity [6]. This molecule consists of three fragments of PGRN and we named it Atsttrin (antagonist of TNF/TNFR signaling via targeting to TNF receptors) [17]. Importantly, Atsttrin has a stronger therapeutic effect than recombinant PGRN in inflammatory arthritis animal models [6]. Recently, another group reported that intraarticular delivery of mesenchymal stem cells (MSCs) which were pretransduced with Atsttrin could protect against OA development [18]. In this study, we examine whether the engineered protein Atsttrin could protect against OA, as well as the molecular mechanisms involved, through the use of human primary chondrocytes in vitro alongside multiple models of OA implemented in genetically modified mice and Sprague–Dawley rats in vivo.

## Methods

### Animals, human cartilage, and recombinant Atsttrin

We performed all animal studies under institutional guidelines. All of the protocols were approval by the Institutional Animal Care and Use Committee, New York University, NY, USA. We generated, maintained, and genotyped the mice with the genetic background of C57BL/6 wildtype (WT), PGRN-deficient (PGRN^–/–^), TNFR1-deficient (TNFR1^–/–^), and TNFR2-deficient (TNFR2^–/–^) mice as reported previously [6]. Sprague–Dawley rats were obtained from Charles River (Wilmington, MA, USA). Eight-week-old male mice and 14-week-old male rats were used for this experiment [19, 20]. For human primary chondrocyte culture, human cartilage samples were harvested from patients receiving total knee joint replacement surgery from New York University, Hospital for Joint Diseases (NY, USA). Acquisition and use of human tissue was conducted in accordance with an Institutional Review Board (IRB#12758) approved protocol. Recombinant Atsttrin was manufactured and provided by Atreaon, Inc.

### Noninvasive anterior crucial ligament rupture rat model

The noninvasive OA rat model was established as described previously [20]. Animals were anesthetized and maintained using isoflurane, and the noninvasive anterior crucial ligament rupture model was established using the indicated machine: Electroforce 3200 (Bose Corp., MN, USA), Solidworks (Dassault Systemes, MA, USA), or Mojo 3D printer (Stratasys, MN, USA). After the model was established, we intraarticularly injected PBS or recombinant Atsttrin once a week for 4 weeks in total. After 4 weeks of treatment, the rats were sacrificed for histological evaluation.

### Surgically induced OA mouse models

For the surgically induced destabilization of medial meniscus (DMM) mouse model, we took advantage of 8-week-old male PGRN^–/–^ mice and their age-matched WT control littermates. The medial meniscotibial ligament of the right knee joint was cut to generate a destabilized medial meniscus. Six mice were used in each group. After surgery, WT mice received local delivery of 6 μl PBS via intraarticular injection, while PGRN^–/–^ mice received local delivery of 6 μl PBS or recombinant Atsttrin (1 μg/μl). Four weeks after model induction, mice were sacrificed and knee joints were collected. The tissues were then processed for histological analysis.

To investigate the preventative as well as the therapeutic effect of Atsttrin, we also established the anterior cruciate ligament transection (ACLT) mouse model. To determine which TNFR was predominantly responsible for Atsttrin’s effect, we established the ACLT mouse model in age-matched WT, TNFR1^–/–^, or TNFR2^–/–^ male mice (*n* = 6, respectively). To address the preventative potential of Atsttrin in OA, we intraarticularly injected 6 μl Atsttrin or PBS once a week for 4 weeks beginning on the day of surgery, as based on our previous study [16]. For examination of Atsttrin’s therapeutic effect, 6 μl Atsttrin or PBS were intraarticularly injected once a week for 4 weeks beginning 4 weeks postoperatively, as based on our previous study [16]. Ambulatory behavior of mice was monitored and recorded throughout the study. After 4 weeks of treatment, mice were sacrificed for dorsal root ganglia (DRG) harvest and histological evaluation.

### Sandwich ELISA for cartilage oligomeric matrix protein

The serum concentration of cartilage oligomeric matrix protein (COMP) was analyzed by our established sandwich ELISA [21]. Protein A agarose (Invitrogen) purified rabbit anti-COMP pAb and anti-COMP type III mAb 2127F5B6 were used as capture and detection antibodies, respectively. Anti-COMP type III mAb 2127F5B6 was labeled with horseradish peroxidase (HRP) using the Lightning-Link™ Horseradish Peroxidase Labeling Kit (Innova) as per the manufacturer’s protocol. Results were interpreted based on the linear range of the standard curve. All of the samples were assayed in triplicate.

### Primary cultures of chondrocytes

Human articular chondrocytes were harvested by enzymatic digestion in accordance with established methodology [16]. Briefly, human cartilage slices were cut into small pieces and washed several times with PBS, pH 7.4. Minced tissues were incubated with agitation in digestion medium comprised of 0.25% collagenase II in DMEM medium with 5% FBS in a spinner flask for 16 hours at 37 °C with 5% CO_2_. After digestion, the suspended cells were collected and seeded into six-well plates for subsequent study. For mouse primary chondrocyte culture, knee joints were collected from 6-day-old WT, TNFR1^–/–^, or TNFR2^–/–^ mice following sacrifice. Under magnification, the cartilage samples were isolated with special attention to avoid damaging the subchondral bone and tissues were rinsed completely three times in PBS. Primary cartilage samples were placed in a 10-cm dish containing the aforementioned digestion buffer and incubated for 16 hours at 37 °C with 5% CO_2_. After full digestion, suspended cells were collected and seeded in a six-well plate for use. All chondrocytes used for experiments are first-generation cells.

### von Frey test

von Frey filaments (Stoelting, Wood Dale, IL, USA) were applied with ascending force intensities on the plantar surface of the hind paw to determine the tactile pain threshold as based on a previous publication [22]. Rapid withdrawal of the hind paw was recorded as a positive response. Hind paws were subjected to 10 trials at a given intensity with a 30-second interval maintained between trials. The number of positive responses for each von Frey filament’s stimulus was recorded. Animals were considered to have reached tactile threshold when five in 10 trials generated a positive response. The examiner was blinded to the groups.

### Dorsal root ganglia isolation

Eight weeks after ACLT surgery, mice were sacrificed and L3–L5 DRG were isolated based on a previous publication [23]. Briefly, mice were anesthetized using isoflurane and fur was cleared from the dorsal surface. A longitudinal incision was made, the spinal column was exposed, and L3–L5 DRG were extracted and tissues flash-frozen using liquid nitrogen. These tissues were processed using the Qiagen RNeasy kit (Qiagen, Valencia, CA, USA) for RNA extraction.

### Luciferase assay

Luciferase assay was performed as reported previously [24]. Lipofectamine2000 DNA transfection reagent was used to cotransfect NF-κB and renilla plasmids in C28I2 cells according to the manufacturer’s protocol (Life Technologies). Eighteen hours after transfection, C28I2 cells were treated without or with 10 ng/ml TNFα in the absence or presence of 200 ng/ml recombinant Atsttrin. After 24-hour incubation, we measured luciferase activity using the Reporter Assay System of Dual-Luciferase® in accordance with the manufacturer's instructions (Promega).

### Histological analysis and immunostaining

Histological analysis was conducted as described previously [16]. Briefly, knee joints were fixed immediately after sacrifice in 4% PFA at room temperature for 48 hours. After washing three times in PBS, the tissues were decalcified at 4 °C in 10% w/v EDTA for 2 weeks. Tissues were measured using a vernier caliper before paraffin processing. Knee joints were dehydrated and embedded; the blocks were trimmed to the midpoint as calculated previously from caliper measurements and serial 5-μm sections were placed on slides for staining. H&E or Safranin O/fast green staining was performed following the established protocol. The extent of synovitis was determined using a graded scale based on H&E staining: grade 0, no signs of inflammation; grade 1, mild inflammation with hyperplasia of the synovial lining without cartilage destruction; and grades 2–4, increasing degrees of inflammatory cell infiltrate and cartilage/bone destruction. For immunohistochemistry staining, sections were pretreated with 0.1% trypsin for 30 minutes at 37 °C. Sections were washed with PBS three times, followed by treatment with 0.25 U/ml chondroitinase ABC (Sigma-Aldrich) for 1 hour and then 1 U/ml hyaluronidase (Sigma-Aldrich) for 1 hour at 37 °C. To reduce nonspecific staining, sections were blocked at room temperature with 20% normal horse serum diluted in 3% BSA for 1 hour. Without washing after blocking, Col X antibody (1:200 dilution; DSHB), MMP-13 antibody (ab3208, 1:200 dilution; Abcam), and affinity-purified monoclonal anti-COMP fragments (1:200 dilution) were diluted in 20% normal horse serum with 3% BSA at 4 °C overnight. Sections were prepared for detection using the Vectastain Elite ABC kit following the manufacturer’s guidelines at 25 °C for 1 hour. Immunorecativity was visualized using 0.5 mg/ml 3,3′-diaminobenzidine (DAB) in 50 mM Tris–HCl substrate, pH 7.8. Methyl green (1%) was used for counterstaining.

### Histological analysis and score

The articular cartilage proteoglycan content was defined on the basis of Safranin O staining. In this study, we used the well-accepted Osteoarthritis Research Society International (OARSI) scoring system [25]: 0 = normal cartilage without any damage; 0.5 = loss of Safranin O staining with no detectable structural change; 1 = small fibrillation; 2 = vertical damage of cartilage limited to superficial layer; 3 = vertical damage, no more than 25% of the cartilage surface; 4 = vertical damage, 25–50% of the cartilage surface; 5 = vertical damage, 50–75% of the cartilage surface; and 6 = vertical damage, more than 75% of the cartilage surface.

### Real-time RT-PCR

Total RNA were extracted from cultured chondrocytes using the RNeasy kit (Qiagen) and reverse transcribed into cDNA using the ImProm-II reverse transcription system (Promega). Data were normalized to the internal control, GAPDH. The primers for specific amplification of murine genes are as follows: 5′-AATGCTGGTACTCCAAACCC-3′ and 5′-CTGGATCGTTATCCAGCAAACAGC-3′ for Aggrecan; 5′-ACTAGTCATCCAGCAAACAGCCAGG-3′ and 5′-TTGGCTTTGGGAAGAGAC-3′ for Col II; 5′-AATCTCACAGCAGCACATCA-3′ and 5′-AAGGTGCTCATGTCCTCATC-3′ for IL-1β; 5′-ACAGGAGGGGTTAAAGCTGC-3′ and 5′-TTGTCTCCAAGGGACCAGG-3′ for NOS-2; 5′-GCATTGACGCATCCAAACCC-3′ and 5′-CGTGGTAGGTCCAGCAAACAGTTAC-3′ for ADAMTS-4; 5′-ACTTTGTTGCCAATTCCAGG-3′ and 5′-TTTGAGAACACGGGGAAGAC-3′ for MMP-13; 5′-CATAGCAGCCACCTTCATTCC-3′ and 5′-TCTCCTTGGCCACAATGGTC-3′ for MCP-1; 5′-AGAGAGCTGCAGCAAAAAGG-3′ and 5′-GGAAAGAGGCAGTTGCAAAG-3′ for CCR-2; and 5′-AGAACATCATCCCTGCATCC-3′ and 5′-AGTTGCTGTTGAAGTCGC-3′ for GAPDH. Melting curve analysis was used to verify the PCR product. Each experiment was repeated three times.

### Western blot analysis

Proteins extracted from chondrocytes were processed on 8% SDS-polyacrylamide gel, followed by electrotransfer to nitrocellulose membrane. The membrane was blocked in 3% BSA in 10 mM Tris–HCl, pH 8.0, 150 mM NaCl, and 0.5% Tween 20. After washing three times, blots were incubated at 4 °C overnight with polyclonal anti-Erk1/2 (#4695, 1:1000 dilution; Cell Signaling Technology), anti-phosphorylated Erk1/2 (#4370S, 1:1000 dilution; Cell Signaling Technology), polyclonal anti-Akt (#9272, 1:1000 dilution; Cell Signaling Technology), anti-phosphorylated Akt (#4058S, 1:1000 dilution; Cell Signaling Technology), polyclonal anti-MMP-3 (ab52915, 1:1000 dilution; Abcam), polyclonal anti-MMP-13 (ab3208, 1:1000 dilution; Abcam), polyclonal anti-ADAMTS-4 (PA1-1750, 1:1000 dilution; Thermo Fisher Scientific), polyclonal anti-NOS-2 (SC651, 1:1000 dilution; Santa Cruz Biotechnology), polyclonal anti-GAPDH (SC25778, 1:1000 dilution; Santa Cruz Biotechnology), polyclonal anti-tubulin (#5346, 1:1000 dilution; Cell Signaling Technology), or diluted polyclonal anti-lamin B (SC-6217, 1:500 dilution; Santa Cruz Biotechnology). After washing three times, blots were incubated with an appropriate HRP-conjugated anti-rabbit/mouse immunoglobulin secondary antibody at 25 °C for 1 hour. The bound antibody was visualized using an enhanced chemiluminescence system (Amersham Life Science, Arlington Heights, IL, USA).

### Cartilage explant cultures

Cartilage explants were cultured as described previously [16]. Briefly, mouse femoral head cartilage was isolated and finely minced to 1 mm diameter and 1 mm thickness. The cartilage explants were then dispensed into serum-free DMEM containing 25 mM HEPES and 2 mM glutamine, in the absence or presence of recombinant Atsttrin (200 ng/ml).

### Dimethylmethylene blue assay of GAG

The mouse cartilage culture medium was collected and GAG release was quantified by dimethylmethylene blue assay (DMMB) (Polysciences, Warrington, PA, USA). Hyaluronidase (0.5 unit/ml; Seikagaku, Tokyo, Japan) was incubated with collected medium for 3 hours at 37 °C to remove hyaluronan in order to reduce inhibition of the DMMB assay. The DMMB signal from digests was measured at 520 nm using a SpectraMax 384 Microplate Reader (Molecular Devices, Sunnyvale, CA, USA). The GAG content was calculated based on linear regression of readings from chondroitin-6-sulfate standards from Shark cartilage (Sigma Aldrich, St. Louis, MO, USA). Each sample was read in triplicate. The average values of the triplicates were normalized to the standard curve.

### Statistical analysis

Results were expressed as mean ± SEM. Statistical analysis included Student’s *t* test performed by SPSS software (SPSS Inc., Chicago, IL, USA). *p* < 0.05 was considered statistically significant.

## Results

### Atsttrin rescues accelerated OA caused by PGRN deficiency

**Fig. 1:**
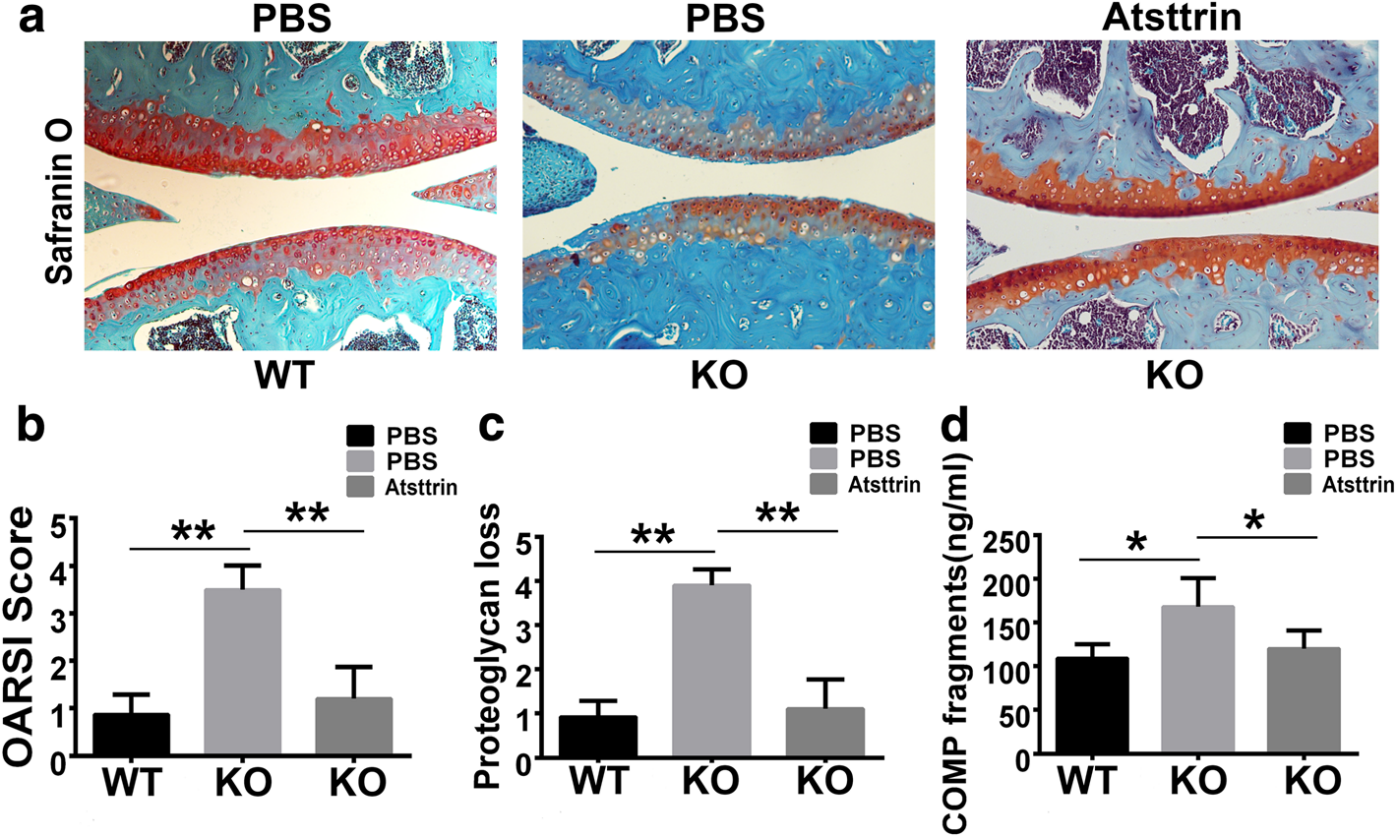
Atsttrin prevented accelerated OA caused by PGRN deficiency. **a** Intraarticular injection of Atsttrin protected against articular cartilage loss following the surgically induced DMM model in PGRN^–/–^ mice, assayed by Safranin O staining. **b, c** Quantification of OARSI score and proteoglycan loss based on Safranin O staining. **d** Atsttrin-reduced serum level of COMP fragments, assayed by ELISA. Values are mean ± SEM of six mice. *p < 0.05, **p < 0.01 versus PBS-treated group. KO knockout, OARSI Osteoarthritis Research Society International, PBS phosphate-buffered saline, WT wildtype

Previously, we reported that PGRN is expressed in both human and mouse articular cartilage, and its level is elevated, relative to healthy controls in both human OA cartilage and in surgically induced OA model mice. In addition, we have reported that loss of PGRN led to enhanced OA in both “aged” mice and surgically induced OA models [16]. In the present study, PGRN expression is slightly, but not significantly, elevated in 1-year-old aged mice as compared with 4-month-old mice (Additional file 1: Figure S1A, B). To determine whether Atsttrin, an engineered protein derived from PGRN [6], could prevent the accelerated OA seen in our surgically induced, PGRN-null OA models, the DMM model was generated in PGRN^–/–^ and age-matched WT mice. As illustrated in Fig. 1a, Safranin O-stained sections demonstrated that Atsttrin effectively prevented loss of proteoglycan content in PGRN^–/–^ mice. Statistical analysis of proteoglycan loss and the OARSI score obtained from Safranin O-stained sections indicates that Atsttrin significantly reduced articular cartilage destruction (Fig. 1b, c). Moreover, as shown in Fig. 1d, application of recombinant Atsttrin also reduced the serum levels of COMP-degradative fragments as assayed by ELISA.

### Atsttrin prevents OA development in surgically induced OA mouse model

**Fig. 2:**
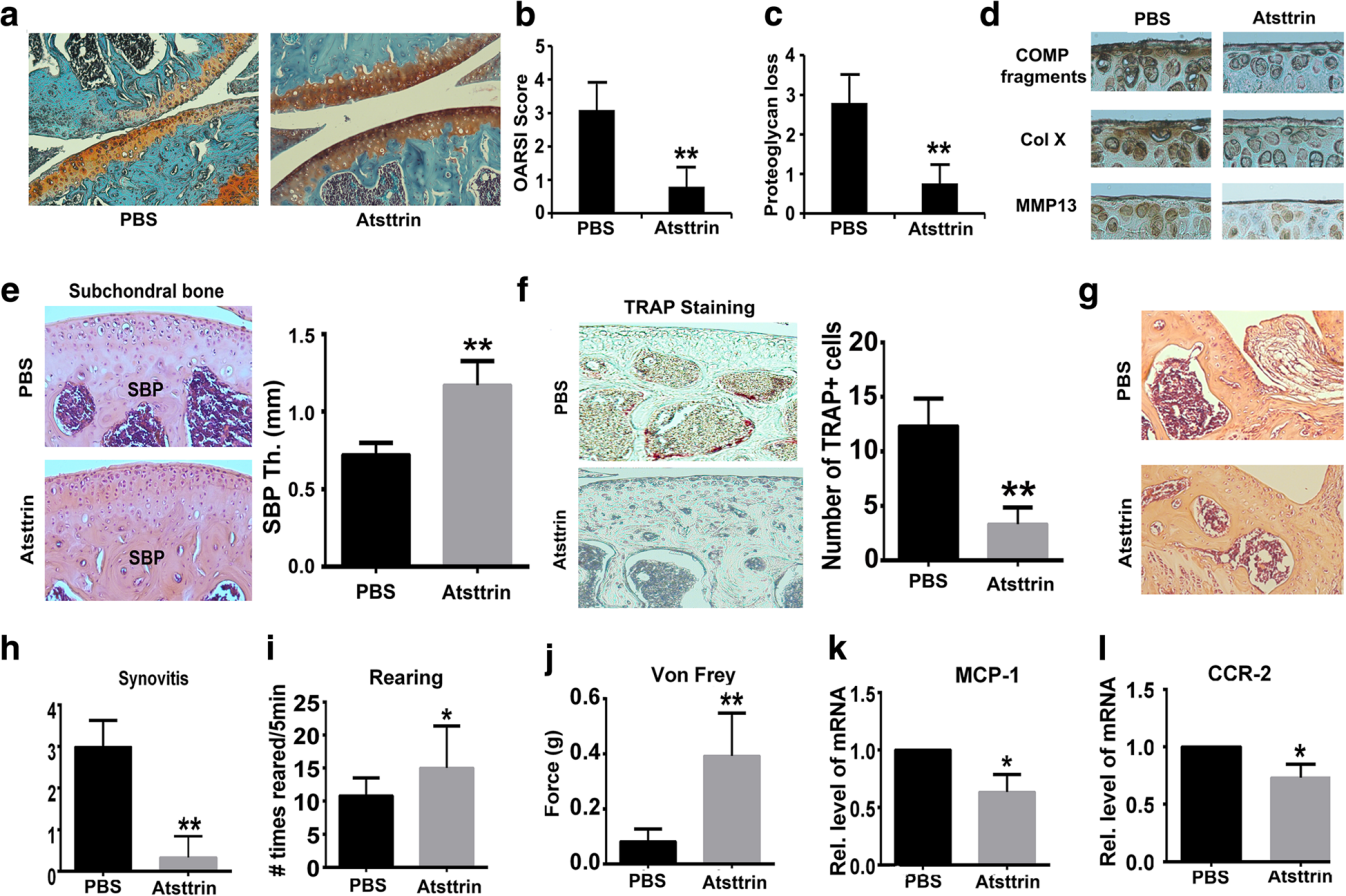
Atsttrin prevents progression of osteoarthritis in vivo in surgically induced OA models. **a** Safranin O-stained sections of ACLT OA model in WT mice. Knee joints collected 4 weeks after the model establishment and processed for Safranin O staining. **b, c** Quantification of OARSI score and proteoglycan loss based on Safranin O staining. **d** Representative photography of immunochemistry stained sections. Positive signal is brown. **e** H&E staining of subchondral bone and quantification of subchondral bone plate thickness. **f** TRAP staining of subchondral bone and quantification of TRAP-positive cells. **g** H&E staining of synovium. **h** Extent of synovitis determined based on H&E staining. **i** Rearing instances within 5 minutes in an open cage. **j** Tactile sensitivity assayed by von Frey testing. **k, l** Transcriptional levels of CCR-2 and MCP-1 in the ipsilateral L3–L5 DRG. Each group composed of six mice. *p < 0.05, **p < 0.01 versus PBS-treated group. CCR-2 chemokine receptor 2, Col X type X collagen, COMP cartilage oligomeric matrix protein, MCP-1 monocyte chemoattractant protein 1, MMP matrix-degrading enzyme matrix metalloproteinase, OARSI Osteoarthritis Research Society International, PBS phosphate-buffered saline, TRAP tartrate-resistant acid phosphatase

Recently, Xia et al. [18] reported that local delivery of Atsttrin-transduced MSCs could effectively protect against OA development in a murine OA model. This finding promoted us to determine whether local delivery of recombinant Atsttrin could prevent OA development. To address this issue, we established the ACLT OA model in C56LB/6 WT mice, followed by intraarticular injection of recombinant Atsttrin (6 μg) once per week for 4 weeks. As shown in Fig. 2a, histological analysis indicated that Atsttrin effectively protected against cartilage loss. Additionally, the OARSI score and proteoglycan loss score based on the histology revealed a significant improvement in the Atsttrin-treated group (Fig. 2b, c). Furthermore, as shown in Fig. 2d, immunohistochemical staining demonstrated that Atsttrin reduced COMP degradation, type X collagen (Col X) expression, as well as matrix-degrading enzyme matrix metalloproteinase 13 (MMP-13) expression. Notably, subchondral bone provides support for articular cartilage [26]. Studies indicate that the thickness of the subchondral bone plate significantly decreases during the first phase of ACLT-induced OA in mice [27] and loss of subchondral bone leads to reduced support and more deterioration of articular cartilage in OA pathogenesis [28, 29]. Interestingly, Atsttrin effectively inhibited subchondral bone loss (Fig. 2e). Moreover, we reported previously that Atsttrin inhibited osteoclastogenesis in vitro [6] and it is believed that osteoclastogenesis is involved in subchondral bone remodeling in ACLT mice [30]. To demonstrate whether Atsttrin-mediated subchondral bone protection occurs through inhibition of osteoclast formation, we performed tartrate-resistant acid phosphatase (TRAP) staining. As illustrated in Fig. 2f, Atsttrin significantly inhibited osteoclast activity. Synovium also plays an important role in the pathogenesis of OA; to further determine the effect of Atsttrin on synovitis, we performed H&E staining and found that Atsttrin effectively inhibited synovial inflammation (Fig. 2 g, h). Behavioral observations and pain marker levels were also recorded to determine whether Atsttrin could reduce OA-related pain. Atsttrin treatment was associated with significantly greater rearing times, as compared to the PBS-treated group when monitored for free ambulation in an open field testing box, as indicated in Fig. 2i. In addition, von Frey filament testing demonstrated that the pain threshold was decreased after ACLT surgery and that Atsttrin effectively improved the pain threshold (Fig. 2j). Further, inflammatory cytokines such as chemokine monocyte chemoattractant protein 1 (MCP-1), chemokine receptor 2 (CCR-2), and interleukin-1β (IL-1β) in DRG play an important role in OA-associated pain. It is reported that upregulated transcriptional levels of MCP-1, CCR-2, and IL-1β in DRG correlate to exhibition of pain-associated behaviors in murine OA models [31]. As indicated in Fig. 2 k, l, the transcriptional level of MCP-1 and CCR-2 in the ipsilateral L3–L5 DRG was significantly upregulated compared to the control group, whereas Atsttrin significantly reduced their expressions to levels comparable to those of the control group. Collectively, these results indicate that Atsttrin is likely to be a disease-modifying and also a symptom-modifying molecule.

### Atsttrin is therapeutic against OA and its therapeutic effects depend on both TNFR1 and TNFR2 pathways

**Fig. 3:**
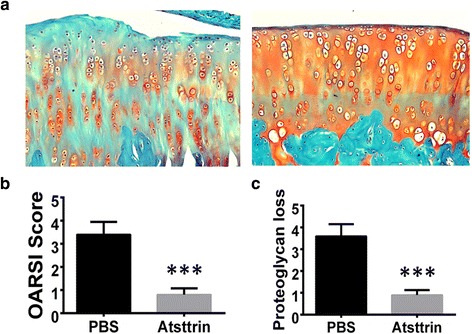
Atsttrin is therapeutic in the noninvasive rat OA model. **a** Safranin O- stained sections of the rat knee joint collected 8 weeks after induction of the noninvasive rat OA model. **b** OARSI score-based Safranin O-stained. **c** Proteoglycan loss score on the basis of Safranin O staining. Each group composed of six rats. ***p < 0.001 versus PBS-treated group. OARSI Osteoarthritis Research Society International, PBS phosphate-buffered saline

To determine whether recombinant Atsttrin is therapeutic against OA, we took advantage of both nonsurgically induced rat and surgically induced mouse models. Four weeks following the establishment of the unique noninvasive rat OA model, we locally delivered 60 μg Atsttrin once per week for 4 weeks. Histological analysis indicated that Atsttrin effectively protected against articular cartilage destruction (Fig. 3a) and significantly improved the arthritis score as well as the proteoglycan loss score (Fig. 3b, c).

**Fig. 4:**
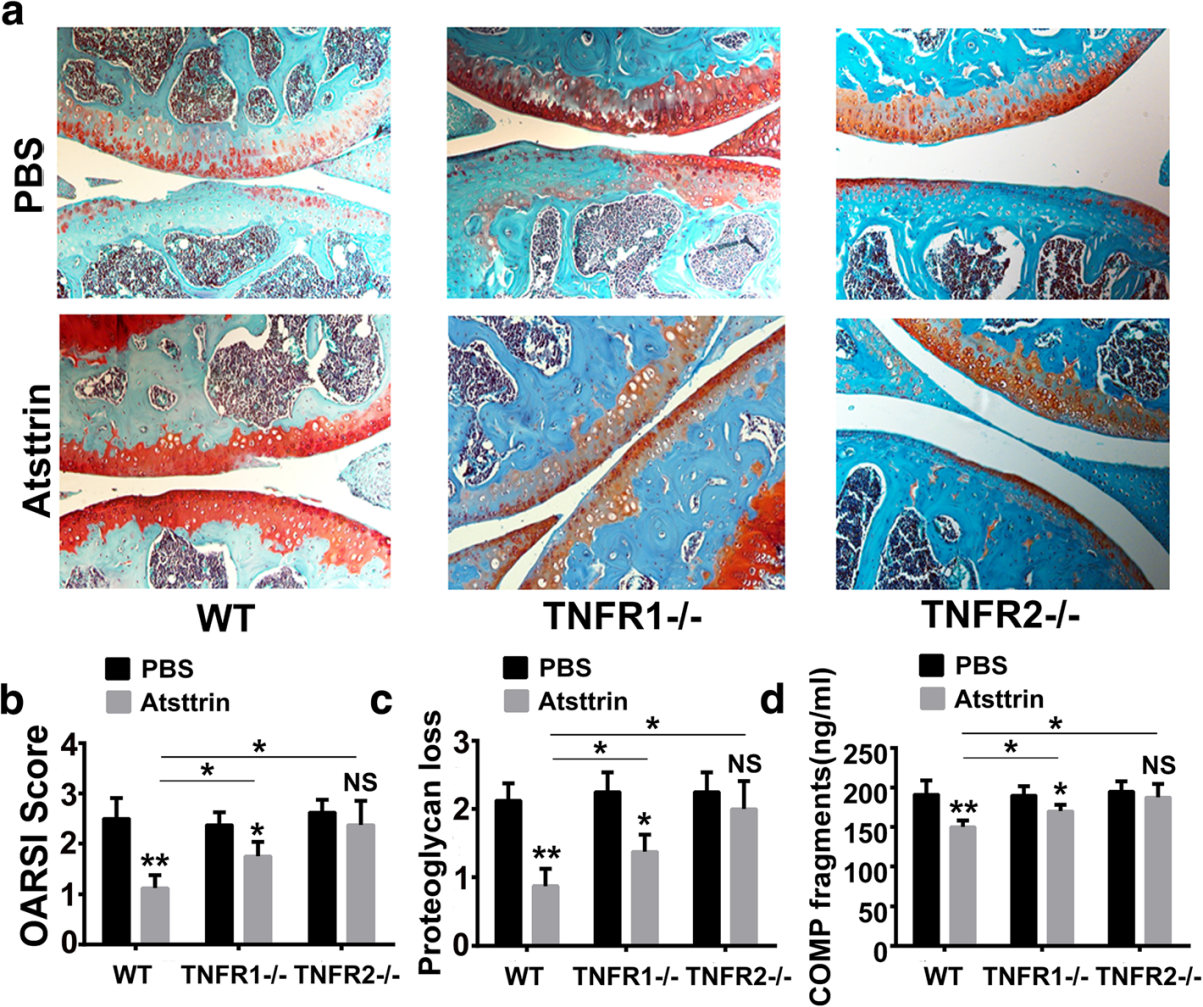
Atsttrin protects against OA through TNFRs. **a** Atsttrin protected against degeneration of articular cartilage in WT mice following surgical induction of the ACLT model, but this protection was partially lost in TNFR1^–/–^ mice and almost abolished in TNFR2^–/–^ mice, assayed by Safranin O staining. *n* = 6 for each group. **b, c** OARSI score and proteoglycan loss based on Safranin O staining. **d** Atsttrin-decreased COMP fragment level in serum, assayed by ELISA. Values are mean ± SEM of at least three independent experiments. *p < 0.05, **p < 0.01 versus PBS-treated group. COMP cartilage oligomeric matrix protein, NS not significant, OARSI Osteoarthritis Research Society International, PBS phosphate-buffered saline, WT wildtype, TNFR tumor necrosis factor receptor

We also took advantage of the well-accepted surgically induced ACLT mouse model. Beginning 4 weeks postoperatively, we intraarticularly injected 6 μg recombinant Atsttrin weekly for 4 weeks. As indicated in Fig. 4a–c, Atsttrin exhibited a therapeutic effect in the WT mice, evidenced by preservation of articular cartilage. To compare the efficacy of PGRN and its derived engineered protein Atsttrin in mitigating OA progression, we also compared the OARSI score between these two groups, and found no significant difference between PGRN and Atsttrin in treating OA (Additional file 2: Figure S2).

To determine whether the Atsttrin-mediated protective effect is dependent upon TNFR1 or TNFR2, or both receptors, we established the ACLT model in TNFR1^–/–^ and TNFR2^–/–^ mice. As revealed in Fig. 4a–c, TNFR1^–/–^ mice exhibited partial loss of Atsttrin-mediated protection while the Atsttrin-mediated protective effect was almost abolished in TNFR2^–/–^ mice. However, we found that, in the presence of Atsttrin, the OARSI score and proteoglycan loss score of both TNFR1^–/–^ mice and TNFR2^–/–^ mice were each significantly increased relative to those of WT mice. Sera from WT mice and from each group of TNFR null mice were collected and COMP fragment-specific ELISA revealed that Atsttrin significantly decreased COMP degradation in the WT OA mice; however, the Atsttrin-mediated protective effect against COMP degradation was slightly decreased in TNFR1^–/–^ mice and relatively unaffected in TNFR2^–/–^ mice (Fig. 4d). Notably, after Atsttrin treatment, the level of COMP fragments in TNFR1^–/–^ mice and TNFR2^–/–^ mice were significantly increased in comparison to levels in WT mice. Collectively, both TNFR1 and TNFR2 appear to be required for mediating Atsttrin’s protective effect in OA.

### Atsttrin-mediated anabolism primarily depends on TNFR2-Akt-Erk1/2 signaling

**Fig. 5:**
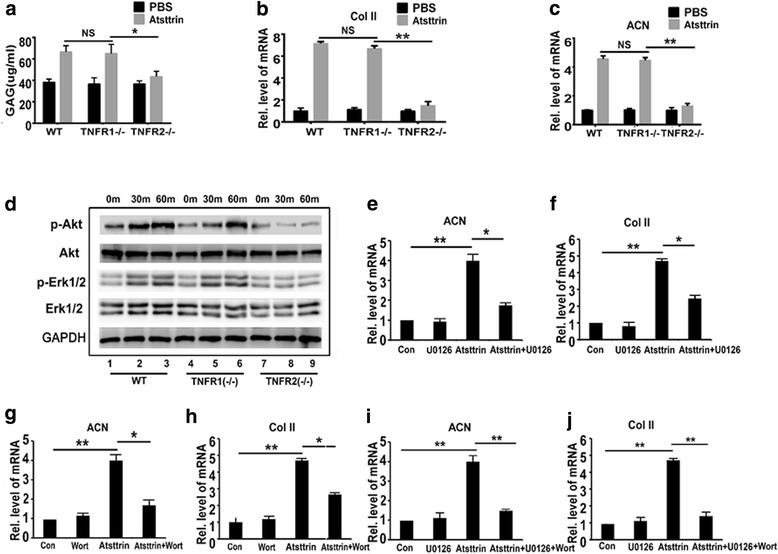
Atsttrin-mediated anabolic effect in chondrocyte primarily depends on TNFR2-Akt/Erk1/2 signaling. **a** Atsttrin-mediated GAG synthesis largely depended on TNFR2. Mouse femoral head cartilage cultured with or without addition of Atsttrin for 7 days, measured by GAG synthesis assay. **b, c** Transcriptional levels of aggrecan (ACN) and type II collagen (Col II). Primary chondrocytes were isolated from WT, TNFR1^–/–^, and TNFR2^–/–^ mice, cultured in absence or presence of Atsttrin, and transcriptional levels measured by real-time PCR. **d** Atsttrin strongly activated Akt signaling and slightly activated Erk1/2 signaling in WT and TNFR1^–/–^ chondrocytes, but lost this activation in TNFR2^–/–^ chondrocytes. **e, f** Transcriptional levels of ACN and Col II in chondrocytes. Chondrocytes were isolated and cultured without or with Atsttrin in absence or presence of 1 μM Erk1/2 signal blocker U0126. **g, h** Transcriptional levels of ACN and Col II in chondrocytes. Chondrocytes were isolated and cultured without or with Atsttrin in absence or presence of 1 μM Akt signaling blocker Wortmannin. **I, j** Transcriptional levels of ACN and Col II in chondrocytes. Chondrocytes were isolated and cultured without or with Atsttrin in absence or presence of 1 μM U0126 and Wortmannin. Values are mean ± SEM of six mice. *p < 0.05, **p < 0.01 versus WT or PBS group. Con control, NS not significant, PBS phosphate-buffered saline, WT wildtype, TNFR tumor necrosis factor receptor, Wort Wortmannin

Given that Atsttrin selectively binds to TNFRs and that the Atsttrin-mediated beneficial effect in OA depends on both receptors in vivo, we next sought to examine the molecular mechanisms involved through detailing the effect of Atsttrin on cartilage and chondrocyte metabolism. To determine whether Atsttrin has an anabolic effect in cartilage, we took advantage of mouse primary cartilage explants and found that recombinant Atsttrin significantly promoted GAG synthesis in WT and TNFR1^–/–^ cartilage while Atsttrin lost this effect in TNFR2^–/–^ cartilage (Fig. 5a). Additionally, Atsttrin-induced expressions of anabolic biomarkers, including type II collagen (Col II) and aggrecan (ACN), were remarkably reduced in TNFR2^–/–^ chondrocytes in comparison with WT and TNFR1^–/–^ chondrocytes (Fig. 5b, c). These data indicate that the Atsttrin-mediated anabolic effect primarily depends on the TNFR2 pathway.

Previous study has shown that both Akt and Erk1/2 pathways are involved in chondrocyte anabolism and PGRN slightly activates Akt signaling and strongly actives Erk1/2 signaling in chondrocytes [13]. Contrastingly, we demonstrate herein that Atsttrin strongly activates Akt signaling and slightly activates Erk1/2 signaling (Fig. 5d). Activation of these signaling pathways was lost in TNFR2^–/–^ chondrocytes, but no change was observed in TNFR1^–/–^ chondrocytes relative to WT chondrocytes (Fig. 5d). More importantly, Atsttrin’s anabolic effect was partially lost after blocking Erk1/2 signaling alone using U0126 or blocking the Akt signaling pathway alone using Wortmannin (Fig. 5e–h). Furthermore, by applying both Erk1/2 and Akt signaling inhibitors simultaneously, we found that Atsttrin-mediated anabolism in chondrocytes was almost abolished (Fig. 5i, j). Taken together, Atsttrin exhibits an anabolic effect in chondrocytes, and this effect primarily relies on TNFR2-Akt-Erk1/2 signaling.

### Atsttrin inhibits TNFα-induced inflammatory catabolism

**Fig. 6:**
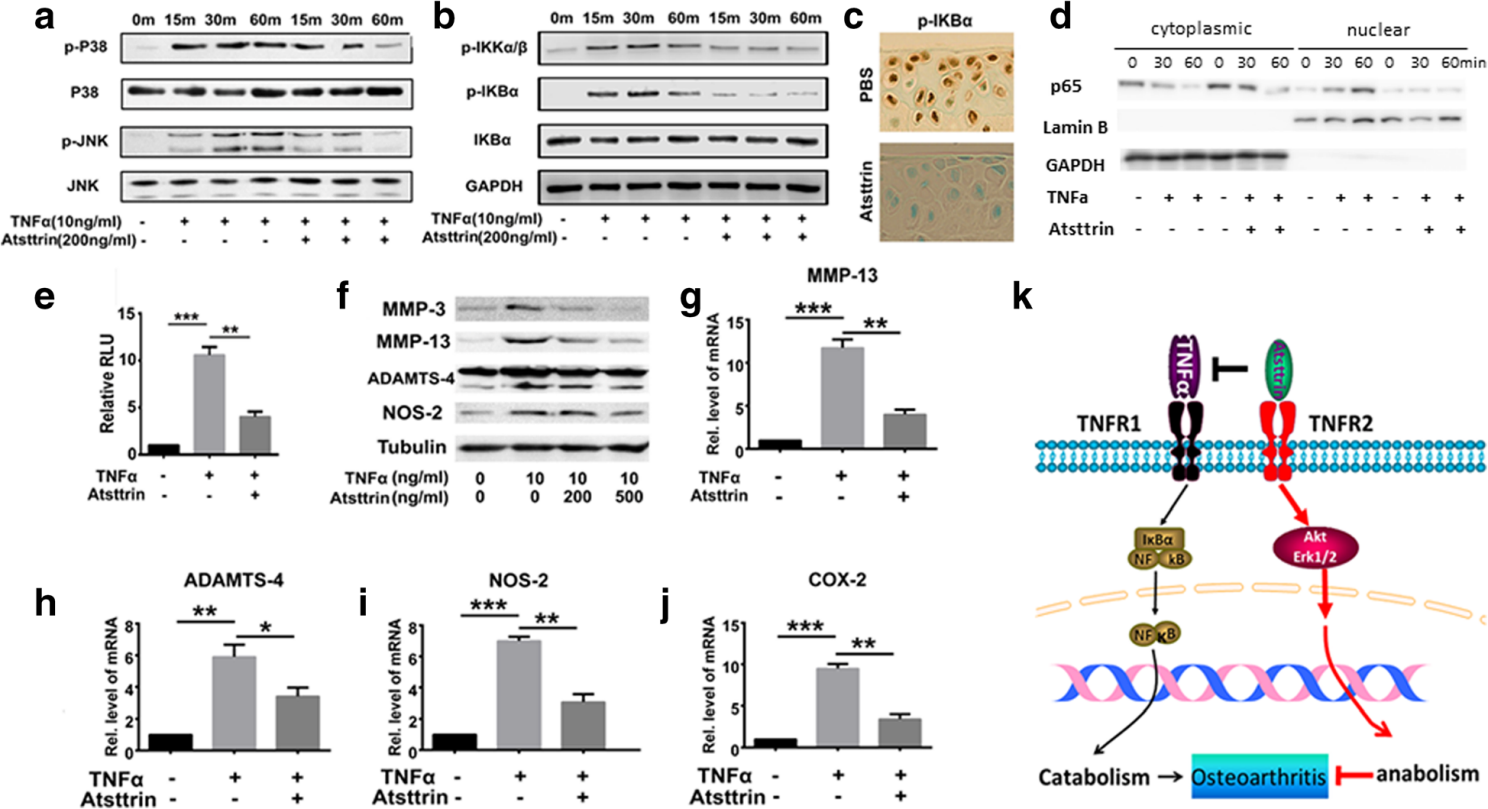
Atsttrin inhibits TNFα-induced catabolic metabolism. **a** Western blot analysis bands of phosphorylation and expression of indicated signaling molecules (p38, p-p38, JNK, p-JNK) at various time points. **b** Human primary chondrocytes cultured without or with 10 ng/ml TNFα in absence or presence of 200 ng/ml Atsttrin for various time points. Phosphorylation and expression of molecules of interest (NF-κB) determined by western blot analysis. **c** Representative image of immunohistochemistry staining for phosphorylated IkBα in the articular cartilage of WT ACLT-model mice. **d** Expression of p65 in primary chondrocyte cytoplasmic or nuclear extracts. Primary chondrocytes cultured without or with 10 ng/ml TNFα in presence or absence of 200 ng/ml Atsttrin. GAPDH and lamin B shown as cytoplasmic and nuclear internal controls, respectively. **e** Luciferase activity of NF-κB reporter gene. **f** Expression of MMP-3, MMP-13, ADAMTS-4, and NOS-2 analyzed by western blot assay. Primary human chondrocytes treated by 10 ng/ml TNFα without or with 200 ng/ml Atsttrin. Tubulin is internal control. **g**–**j** Transcriptional levels of catabolic inflammatory biomarkers, including *MMP-13*, *ADAMTS-4*, *NOS-2*, and *COX-2*, in primary human chondrocytes. Values are normalized mean ± SEM. *p < 0.05, **p < 0.01 versus control group. Six cartilage samples used in each group. **k** Proposed model for demonstrating Atsttrin’s role and mechanism in OA. MMP matrix-degrading enzyme matrix metalloproteinase, PBS phosphate-buffered saline, TNFR tumor necrosis factor receptor, TNFα tumor necrosis factor alpha

It is known that TNFα plays an important role in OA [32]. Additionally, TNFα could activate MAPK and NF-κB signaling [6], subsequently inducing matrix-degrading enzymes, including MMP-13 and ADAMTS-4, as well as other inflammatory biomarkers, such as NOS-2, in chondrocytes. The finding that Atsttrin protected against TNFα-induced cartilage loss in multiple rheumatoid arthritis mouse models promoted us to determine the interplay between TNFα and Atsttrin in OA. Human primary chondrocytes were cultured in the absence or presence of 10 ng/ml TNFα with or without 200 ng/ml Atsttrin for various lengths of time. Western blot analysis indicated that Atsttrin inhibited TNFα-activated signaling pathways (Fig. 6a). Since TNFα exerts its inflammatory action largely through activation of classical NF-κB signaling [6], we next examined whether Atsttrin altered TNFα-mediated NF-κB phosphorylation, translocation, and activity. As elucidated in Fig. 6b, Atsttrin effectively inhibited phosphorylation of NF-κB in primary human chondrocytes in vitro. Additionally, immunochemistry staining demonstrated that Atsttrin dramatically inhibited NF-κB phosphorylation in the articular cartilage of mice induced under the ACLT model (Fig. 6c).

Separately extracting cytoplasmic and nuclear protein from human primary chondrocytes also revealed differences in NF-κB translocation in treated and untreated cells. As illustrated in Fig. 6d, without TNFα treatment p65 was mainly detected in the cytoplasm; after TNFα treatment, cytoplasmic p65 was decreased and nuclear p65 was increased. In contrast, TNFα-mediated NF-κB nuclear translocation was nearly abolished in the presence of Atsttrin. We next transfected a NF-κB reporter gene into C28I2 human chondrocyte cells (provided by Dr Mary Goldring, Hospital for Special Surgery, New York, NY, USA [33]) to test whether Atsttrin altered NF-κB activity. As shown in Fig. 6e, Atsttrin significantly inhibited TNFα-induced NF-κB activity. To further elucidate the mechanism, we cultured primary human chondrocytes in the absence or presence of 10 ng/ml TNFα with or without 200 ng/ml Atsttrin for 6 hours. After this, the total RNA was extracted for real-time PCR analysis. As indicated in Fig. 6 g–j, Atsttrin significantly decreased TNFα-upregulated transcriptional levels of MMP-13, ADAMTS-4, NOS-2, and COX-2. After 48-hour incubation, Atsttrin exhibited inhibition of TNFα-induced protein levels of inflammatory matrix-degrading enzymes and inflammatory cytokines in a dose-dependent manner (Fig. 6f).

## Discussion

OA is one of the most common joint diseases; however, the exact pathological mechanism of OA is still largely unclear [34]. Unfortunately, no drugs are able to prevent or halt the progression of OA [35]. Regardless of the complicated etiology of OA, it is well accepted that cytokines are closely involved in initiating and aggravating OA. Our genome-wide screen found that PGRN was an OA-related growth factor [6]; levels of PGRN were also significantly elevated in the joints of arthritic patients [14]. The finding that PGRN deficiency accelerated OA while recombinant PGRN ameliorated OA prompted us to determine whether the PGRN-derived engineered protein Atsttrin could rescue enhanced OA brought on by PGRN deficiency. In the present study, we took advantage of the DMM model in WT and PGRN^–/–^ mice and found that Atsttrin effectively prevented PGRN deficiency-mediated OA, evidenced by less cartilage destruction and reduced serum level of COMP fragments. In our previous study, we found that, compared with WT littermates, PGRN deficiency led to more severe synovium inflammation as well as osteophyte formation. In line with this finding, Atsttrin could effectively reduce synovium inflammation. However, we failed to observe osteophyte formation in our current model in either PBS-treated or Atsttrin-treated mice, which may be attributable to the duration of our model. Observation and analysis of the osteophyte formation phenotype may require an extended time course.

The surgically induced mouse model is a well-accepted method to investigate OA pathogenesis in vivo [36], whereas the noninvasive rat model can mimic closed-joint injury-induced OA [20, 37]. Our finding that intra-articular delivery of recombinant Atsttrin could dramatically prevent cartilage destruction in both mouse and rat OA models is in line with reports that local delivery of Atsttrin-transduced MSCs is protective against OA-related cartilage destruction in vivo [18]. OA is also characterized by progressive loss of extracellular matrix, leading to the breakdown of articular cartilage [38]. COMP is a noncollagenous molecule of the extracellular matrix in articular cartilage and plays an important role in maintaining chondrocyte function and cartilage integrity. COMP is fragmented upon degradation and the elevated serum level of COMP fragments observed in the progression of OA is considered a biomarker of disease activity [39]. Previously, we showed that Atsttrin dramatically inhibited COMP degradation in animal models of inflammatory arthritis [6]. In the current study, we found that Atsttrin also prevented COMP degradation in OA progression. Furthermore, Atsttrin dramatically reduced MMP-13 expression, which is significantly increased in OA and is thought to be the major enzyme responsible for digesting major cartilage component Col II.

Underlying articular cartilage, subchondral bone provides nourishment for cartilage and subchondral bone deterioration is often observed alongside cartilage defects [40]. Previous reports demonstrate that the thickness of subchondral bone gradually decreases during the early phase of the ACLT mouse model; in the late phase, the thickness of subchondral bone gradually increases but does not return to a thickness representative of a normal joint [27]. Additionally, studies have indicated that molecules targeting subchondral bone demonstrate a therapeutic effect in OA [30]. In the present study, we found that Atsttrin effectively inhibited subchondral bone loss. Notably, osteoclastogenesis plays an important role in subchondral bone remodeling in OA [30] and we have shown previously that Atsttrin significantly inhibited osteoclastogenesis in vitro [6]. Here, we report that Atsttrin also inhibited osteoclast activity in OA progression, ultimately lending to preservation of subchondral bone.

Besides pathological changes, the OA-related pain and biomechanical dysfunctions are the major complaints of patients [41] and OA pain is associated with pathological structural severity [42]. It is believed that anti-inflammatory drugs could reduce pain in the pathogenesis of OA [43, 44]. Herein, we found that Atsttrin effectively relieved OA-associated pain by improving travel distance and tactile sensitivity in mice with experimental OA. Additionally, alteration of pain markers and inflammatory molecules in the sensory neurons of the DRG has been reported as a result of interactions between neuropathic pathways and OA tissues [22]. Here we found the levels of mRNA for proinflammatory cytokines in DRG were also significantly suppressed by Atsttrin in OA progression. Besides, a recent study indicated that PGRN could attenuate pain by binding to ATG12 and regulating autophagy [45]. Whether Atsttrin also functions through this mechanism needs further investigation.

There are two distinct receptors for TNFα: TNFR1 and TNFR2 [46]. TNFα/TNFR1 signaling is the classical pathway and is thought to mediate inflammatory signaling [47]. On the contrary, TNFR2 signaling is still largely unknown [8]. Recent studies indicate that TNFR2 activates protective and proliferative pathways [48, 49]. Specifically, studies indicate that TNFR2 effectively protects against TNFα-mediated heart failure [50]. Additionally, TNFR2 has been shown to promote cancer growth [51]. Furthermore, TNFR2 was found to be required for PGRN-mediated immune regulation, cartilage homeostasis, physiological bone formation, and inhibition of LPS-induced lung inflammation [52]. Atsttrin exhibited much higher affinity for TNFR2, when compared to TNFα [53]. Here, we found that Atsttrin’s protective effect against OA primarily depends on TNFR2, although inhibition of TNFR1 inflammatory signaling also partially accounts for Atsttrin’s therapeutic action in OA.

It is known that Erk1/2 and Akt signaling are involved in chondrocyte protection, and that PGRN slightly activates Akt signaling and strongly actives Erk1/2 signaling [13]. In addition, PGRN-mediated chondroprotective effect primarily depends on TNFR2-Erk1/2 signaling [16]. Although Atsttrin is derived from PGRN, it exhibits distinct signal activation patterns. In the present study, we found that Atsttrin strongly activates Akt signaling whereas Erk1/2 signaling is only slightly activated. This signaling activation was lost in TNFR2-deficient chondrocytes but maintained in TNFR1-deficient chondrocytes, which implies that TNFR2 plays a major role in mediating Atsttrin’s proanabolic effects. Furthermore, after applying specific inhibitors of Akt and Erk1/2 signaling, Atsttrin completely lost its proanabolic effect. Thus, together with in-vivo data, we found that the Atsttrin-mediated anabolic effect in OA depends on TNFR2-Akt/Erk1/2 signaling.

As an important proinflammatory cytokine, upregulated TNFα directly promotes inflammatory reactions and triggers chondrocyte death in OA [54]. Furthermore, TNFα-induced metalloproteinases, such as ADAMTS-4, ADAMTS-7, as well as ADAMTS-12, degrade cartilage matrix, leading to deteriorative articular cartilage [55]. TNFα inhibitors demonstrate a therapeutic effect in murine models [56] and OA patients in clinical trials [57, 58]. Additionally, a report from another group showed that Atsttrin promoted bone healing through TNF/TNFR signaling [59]. In the present study, the Atsttrin-mediated chondroprotective effect occurred partially through TNFR1 in vivo. Additionally, a mechanistic study demonstrated that Atsttrin effectively inhibited TNFα-induced inflammatory catabolism in OA progression. Specifically, Atsttrin effectively inhibited TNFα-induced NF-κB phosphorylation, translocation, and activity and the expression of inflammatory catabolic markers such as NOS-2, COX2, as well as ADAMTS-4. Intriguingly, a recent study revealed that DR3, the highest homolog of TNFR1 in the TNFR family, is also capable of binding Atsttrin [60]. TNF-like ligand 1A (TL1A) is the sole identified ligand for DR3. Importantly, Atsttrin could inhibit the interaction between DR3 and TL1A [60]. Thus, whether the Atsttrin-mediated chondroprotective effect partially depends on the DR3 pathway needs to be further investigated.

Although Atsttrin was shown to be more effective than PGRN in preventing inflammatory arthritis [6, 10], we did not find a significant difference between PGRN and Atsttrin in terms of protecting against OA development. Based on our previous studies, we surmise that this disease specificity results from the fact that Atsttrin has a better anti-inflammatory/anti-catabolic TNFα/TNFR1 effect, whereas PGRN has a better effect in activating the anabolic TNFR2 pathway.

It is noted that we used a similar OA model with the 2-month-old mice as we did in our previous publication [16]. Atsttrin’s long-term chondroprotective effect in various animal models, including DMM model mice 4–6 months old and "aged" PGRN-deficient mice that spontaneously develop an OA-like phenotype [16], warrants further investigation. We have shown that Atsttrin rescued the accelerated surgically induced OA phenotype seen in PGRN-deficient mice (Fig. 1) and we anticipate that Atsttrin would also be effective in preventing spontaneous OA in "aged" PGRN-deficient mice.

## Conclusions

Based on the present study and previous publications, a model was proposed (Fig. 6 k). This model illustrates that Atsttrin plays a chondroprotective role in OA through at least two pathways. Atsttrin directly binds to TNFR2 to activate the anabolic signaling pathway; additionally, Atsttrin competitively binds to TNFR1 to inhibit TNFα-mediated inflammatory and catabolic reactions. However, it is unclear whether crosstalk exists between these two pathways. For instance, whether inhibition of downstream NF-κB signaling (via TNFR1) by Atsttrin would mechanistically lead to improvement of the chondrocyte phenotype has not yet been addressed. In addition, the downstream mediators of TNFR2-Erk/Akt activation by Atsttrin also remain largely unknown. These unsolved issues warrant further investigation. In summary, these findings not only provide new insights into the role of PGRN and its derived engineered protein Atsttrin in cartilage homeostasis as well as OA in vivo, but may also lead to new therapeutic alternatives for OA as well as other relative degenerative joint diseases.

## Additional files

Additional file 1: Figure S1. showing expression of PGRN in cartilage at different ages, assayed by immunohistochemistry

Additional file 2: Figure S2. showing quantification of OARSI score based on Safranin O staining for PBS, PGRN, or Atsttrin-treated ACLT mice (DOCX 56 kb)

## Abbreviations

ACLT: Anterior cruciate ligament transection
ACN: Aggrecan
Atsttrin: Antagonist of TNF/TNFR signaling via targeting to TNF receptors
CCR-2: Chemokine receptor 2
Col II: Type II collagen
Col X: Type X collagen
COMP: Cartilage oligomeric matrix protein
DMM: Destabilization of medial meniscus
DRG: Dorsal root ganglia
ELISA: Enzyme-linked immunosorbent assay
HRP: Horseradish peroxidase
IL-1β: Interleukin-1β
MCP-1: Monocyte chemoattractant protein 1
MMP-13: Matrix-degrading enzyme matrix metalloproteinase 13
OA: Osteoarthritis
PCR: Polymerase chain reaction
PGRN: Progranulin
TNFR: Tumor necrosis factor receptor
TNFα: Tumor necrosis factor alpha
TRAP: Tartrate-resistant acid phosphatase
