# Supplementary material for: Progranulin derivative Atsttrin protects against early osteoarthritis in mouse and rat models
Source: Arthritis Res Ther. 2017 Dec 19;19:280. doi: 10.1186/s13075-017-1485-8 (PMC5735869; doi:10.1186/s13075-017-1485-8)
Supplement: Supplementary file 1 — showing expression of PGRN in cartilage at different ages, assayed by immunohistochemistry [file 13075_2017_1485_MOESM1_ESM.docx]

**Additional file 1**


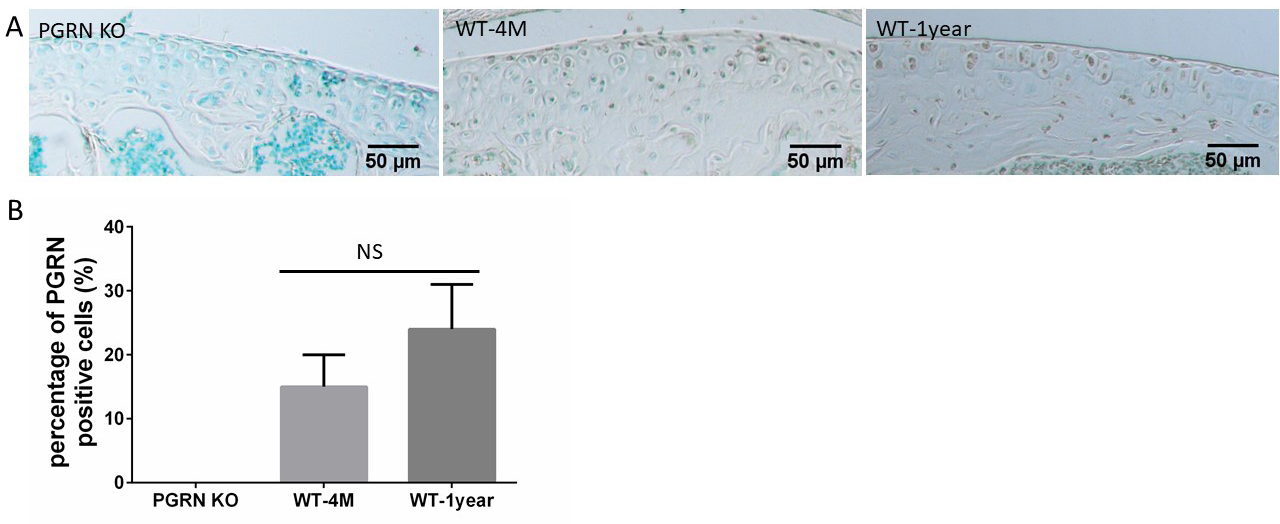


**Supplementary Fig. S1. Expression of PGRN in cartilage at different age, assayed by immunohistochemistry.** (A) Femur cartilage sections were stained with anti-PGRN (brown) antibody and counterstained with methyl green. Section obtained from PGRN KO mouse is served as negative control. Scale bar=50µm. (B) Level of PGRN in mice cartilage at different age was determined by counting the percentage of immunohistochemistry staining positive cells over total cells (n=4). ns = no significance
