# Supplementary material for: Progranulin derivative Atsttrin protects against early osteoarthritis in mouse and rat models
Source: Arthritis Res Ther. 2017 Dec 19;19:280. doi: 10.1186/s13075-017-1485-8 (PMC5735869; doi:10.1186/s13075-017-1485-8)
Supplement: Supplementary file 2 — showing quantification of OARSI score based on Safranin O staining for PBS, PGRN, or Atsttrin-treated ACLT mice (DOCX 56 kb) [file 13075_2017_1485_MOESM2_ESM.docx]

**Additional file 2**


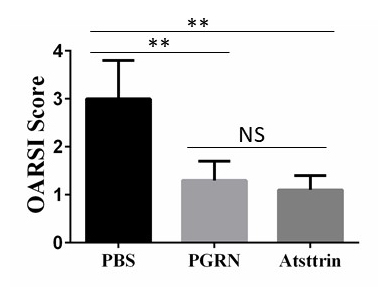


**Supplementary Fig. S2. Quantification of OARSI score based on Safranin O staining for PBS, PGRN or Atsttrin treated ACLT mice (n=6 for each group).** WT mice were established with ACLT OA model, followed by intra-articular injection of PBS, PGRN or Atsttrin. Values are the normalized mean±SEM. NS=No significant difference, ** p<0.01
